# A Possible New Field in Surgery

**Published:** 1886-11

**Authors:** 


### EDITORIAL.

A POSSIBLE NEW FIELD IN SURGERY.

An editorial in a recent number of the *Medical Record* contains some very interesting observations on the organization of foreign bodies within animal tissues. The conversion of bone drainage-tubes, and even sponge, into organized tissues has been observed repeatedly, but these substances are all highly organized, and that they may receive a blood-supply and become connected with the surrounding tissues is not surprising.

The article quoted by the *Record* is from the pen of M. Vanlair in the *Revue de Chirurgie*, and relates to the organization of a rubber tube used in some experiments in the tube suture of nerves. The tube was of ordinary gray caoutchouc, having been in position for seven months and a half. The upper end of the tube was intact, and it gradually tapered downward until it was finally lost in the surrounding tissues; a portion of the tube having evidently undergone partial absorption.

On microscopical examination the rubber was found to have lost entirely its amorphous appearance, and to have given place to a rather dense connective-tissue framework, within the interfasicular spaces of which were found irregular cells of about twenty micro millimeters diameter, with a large nucleus, containing one or more nucleoli. The capillary network was extensive and, in many places, presented the ordinary endothelial lining. At its lower part the tube had become assimilated to the surrounding connective-tissue, and had finally disappeared.

The *Record* observes in conclusion: "That india rubber can thus become organized is the more remarkable when we consider that it is a pure vegetable exudation, devoid of all structure, and seemingly more calculated to act as a foreign body and to prevent the union of wounded surfaces than to undergo organization and to become thus an integral part of the animal tissues."

In a report read before the California State Dental Society,* Dr. William J. Younger describes the following case of implantation of a tooth: "In the early part of March, 1886, a lady, the wife of a well-known dentist, brought me a bicuspid that had been extracted at her solicitation, in Sacramento, on the thirty-first day of January, 1885, in the belief that it was the seat of a neuralgic pain, which had been the cause of great anguish to her. This tooth, brought to me after this long lapse of time, had in the meanwhile been carried about in her portemonnaie, stowed away in her jewel case, and shuffled about in her bureau drawer, and this tooth she wanted replanted in her jaw! My first impulse was to laugh, my next to argue with her about the impossibility of success of such an operation, explaining to her that it was due only to the vitality of the membrane covering the root that the operation owed its success; that without this living membrane the tooth was as impossible of attachment as so much bare ivory or porcelain."

The doctor finally consented to do the operation as an experiment; accordingly, on the 11th day of March, 1886, a socket was drilled, and after soaking the tooth in warm water for about twenty-five minutes it was replaced in the position from which it had been taken thirteen months and eleven days previously. The tooth became firmly fixed. Since this first experiment, teeth that had been extracted for months have

---

* Abstract *Weekly Medical Review.*

been implanted, and with such success that the procedure will probably become one of the recognized operations of dental surgery.

While a few isolated facts of this kind prove but little, yet when added to previous observations of a similar nature they make a substantial advance in our knowledge. The various operations of plastic surgery, the skin and sponge-grafting, and even the *bizärre* operation of sheepskin-grafting attempted in the Cook County Hospital a few years ago, all suggest the possibilities of the surgery of the future, with additional knowledge of the substances which are most readily assimilated and easiest to become integrals of animal tissues; the best methods of proceeding in the application of such substances, and the manner in which their vitality is maintained, and connections made with previously formed tissues. In the light of the above facts one may not be going beyond proper limits in the use of "scientific imagination" in predicting that the time may come when in many more cases the connective-tissue corpuscles necessary in the repair of all tissues may be supplied from other sources than that of the chylopoietic system of the patient.

The immunity from wound-infection already secured by modern antiseptic methods, and their seeming influence on the healing process would appear to leave but little to be desired. That other advances may yet be made in the direction indicated, in the future, seems not improbable.

